# Adaptation of the Consultation and Relational Empathy Measure to Turkish

**DOI:** 10.3390/bs15060721

**Published:** 2025-05-23

**Authors:** Murat Erzurumlu, Habibe Özçelik, Melahat Akdeniz, Ethem Kavukçu, Hasan H. Avcı

**Affiliations:** 1 Department of Family Medicine, School of Medicine, Akdeniz University, Antalya 07058, Turkey; dr.murat.erzurumlu@gmail.com (M.E.); melahatakdeniz@akdeniz.edu.tr (M.A.); 2 Department of Public Health Nursing, Faculty of Nursing, Akdeniz University, Antalya 07058, Turkey; hozcelik@akdeniz.edu.tr; 3 Department of Sports Medicine, School of Medicine, Akdeniz University, Antalya 07058, Turkey; ethemkavukcu@gmail.com

**Keywords:** empathy, consultation, medical doctor, patients, reliability, validity

## Abstract

The Consultation and Relational Empathy (CARE) measure was developed to create a means of evaluating a physician’s consultation process based on an extensive and meaningful definition of empathy, independent of a patient’s socioeconomic background. The aim of this study was to adapt the valid and reliable CARE measure into Turkish and to confirm the measure’s validity and reliability in the Turkish language. The measure reached its final version after the processes of translation, back-translation, and a pilot study. The final version of the measure was administered to 300 individuals between the ages of 18 and 83 who were patients at Akdeniz University Hospital. The internal consistency, homogeneity, and construct of the measure were assessed. The internal consistency and homogeneity of the measure were found to be very high (Cronbach’s alpha = 0.973; the corrected item–total correlation coefficient for all items > 0.816). The measure was found to have a single-factor structure with a high level of construct validity. It was additionally found that the CARE score was not affected by the patients’ demographic characteristics. The Turkish version of the CARE measure can be used as a valid and reliable measuring tool. The CARE measure is a tool with which patients can assess the level of empathy shown by a healthcare provider.

## 1. Introduction

Empathy is an inevitable part of a consultation with a physician and also directly affects all healthcare service processes. Empathy not only plays a role in the communication between patient and physician but is also connected to each step of the healthcare process from diagnosis to treatment, influencing the achievement of patient and physician satisfaction, patient compliance, the prevention of long-term complications, increasing quality of life, and reducing mortality rates (Decety & Fotopoulou, 2015; Hojat et al., 2011; Rakel et al., 2009; Smith et al., 2016). Consequently, the ability to harbor empathy must be analyzed in terms of every dimension of the health sector, and an effort must be made to increase the bounds of this capability.

While the term “empathy” has been defined in various ways in the fields of the social sciences and humanities, it remains the most important phenomenological concept that contributes to people’s understanding of the person or object before them (Montag et al., 2008). Empathy, according to DSM-5, refers to the ability to understand and appreciate other people’s experiences and motivation, to show tolerance towards different perspectives, and to become aware of the impact of one’s own behavior on others (American Psychiatric Association, 2013). The Turkish Language Society offers the word “duygudaşlık” as the corresponding Turkish word for “empathy” (Empati TDK Sözlük Anlamı, 2024). The Society of General Internal Diseases has defined “empathy” (“empati” in Turkish) as the ability to understand another person’s emotional state without actually going through the experience oneself (Markakis et al., 1999).

In the field of medicine, empathy is considered to be one of the fundamental values of humanitarian medicine, one that provides many benefits to both patients and healthcare professionals (Decety, 2020; Reynolds & Scott, 1999). It has been shown that establishing an empathic relationship has a positive influence on communication between patient and physician (Mercer & Reynolds, 2002). A sense of empathy facilitates the decision-making process shared by the patient and physician. At the same time, empathy is important for being able to make an evaluation from a broad perspective (Dambha-Miller et al., 2019; Wensing et al., 1998). Patients who are shown an empathetic approach are known to display increased patient satisfaction (Little et al., 2015; Soltner et al., 2011) and a marked enhancement in the respect and trust they have for their doctors (Beckman et al., 1994; Levinson et al., 1997).

The role of empathy in clinical processes is not restricted to communication and directly affects clinical practices as well. Various articles in the literature have reported that an increase in the empathy score of health providers is responsible for lowering cardiovascular event risks, mortality rates (Dambha-Miller et al., 2019), and patients’ pain scores (Chassany et al., 2006), as well as for achieving improvements in blood glucose and cholesterol levels in patients with diabetes (Hojat et al., 2011) and drops in the severity and duration of symptoms in patients diagnosed with influenza (Rakel et al., 2009). It is due to these factors that there is a need for a measure that will facilitate the assessment of empathy (Mercer et al., 2005). Such tools can be divided into three groups: those that assess the level of empathy as evaluated by health professionals themselves and those that assess this trait as evaluated by an observer or the patient (Pedersen, 2009). Many measures that assess the level of empathy of health professionals are mentioned in the literature, but these are generally based on questionnaires that are answered by healthcare providers themselves (Pedersen, 2009). This raises the concern that healthcare providers may be influenced by the education that they were provided about empathy or that they may not be reflecting patients’ views in their assessments (Mercer & Reynolds, 2002). However, the Consultation and Relational Empathy (CARE) measure (Kane et al., 2007) is a tool with which patients can assess the level of empathy shown by a healthcare professional in primary, secondary (Mercer & Murphy, 2008), and tertiary healthcare services (Park et al., 2022). Since the CARE measure was developed, the tool has been translated into many languages, including Chinese, Croatian, Dutch, Swedish, Portuguese, Hindi, Spanish, and Italian (Aomatsu et al., 2014; Crosta Ahlforn et al., 2017; Fung et al., 2009; García Del Barrio et al., 2021; Hanževački et al., 2015; Manzoni et al., 2019; Natali et al., 2022; van Dijk et al., 2017). Although there are measures in Turkish that have been designed to assess a physician’s level of empathy according to the physician’s responses to a questionnaire (Gönüllü & Öztuna, 2015), there is still a need for a tool that will help to evaluate a physician’s empathy level from the perspective of the patient. The CARE measure assesses physicians’ empathy levels from the patients’ perspective.

The aim of this study was to undertake the first adaptation of the CARE measure into Turkish, test the Turkish version for validity and reliability, and discover whether the results are at all influenced by demographic factors such as age or gender.

## 2. Materials and Methods

Our study was accordingly planned as a methodological investigation into the validity and reliability of the Turkish version of the CARE measure.

### 2.1. Translation of the CARE Measure into Turkish

Prior to the adaptation, the written consent of the developer of the CARE measure, Stewart MERCER, was obtained for the validity study. The first step entailed the translation of the CARE measure into Turkish by four independent Turkish-speaking translators who were fluent in English (two doctors, one public health nurse academic, and one independent translator). The second step involved the comparison of the two texts and agreeing on a draft of the best wording of the translation of each item. In the third step, a panel of 5 experts experienced in the adaptation of measurement tools and fluent in both languages reviewed the scale content. After the expert review and scoring, the Content Validity Index (CVI) was calculated and found to be high (1.00). In the fourth step, two translators fluent in both languages and who were not privy to the original text and not involved in the translation work back-translated the text into English independently of each other. The fifth step entailed the comparison of the back-translation produced in Step 4 with the original measure. Following this reevaluation, the Turkish version was appropriately revised. The sixth step consisted of a pilot implementation of the draft measure with 37 individuals who matched the sample requirements. The people participating in the pilot study were queried as to the clarity and comprehensibility of the items and whether they were able to fully understand the statements. Lastly, the Turkish version was given its final form, thus producing the Turkish adaptation of the CARE measure.

### 2.2. Study Setting and Sampling

The study participants were selected using a simple randomized sampling method from individuals who had presented to the Akdeniz University Hospital for an examination over the period of October–November 2022. The participants were first invited to a predesignated area for data collection and informed about the nature of the study. After providing their informed consent, the participants filled out the data collection forms under the supervision of the researcher; completing the forms took an average of 5–8 min.

The inclusion criteria for the study were determined as follows: being 18 or older, consenting to participate in the research, having had an examination in the last 24 h, and being a native Turkish speaker. Participants were excluded from the study if they had a chronic, mental, and/or cognitive disease, did not understand the CARE measure, wished to withdraw from the study, were not fluent in Turkish, or had 3 or more missing data in the questionnaire.

There are several commonly followed methods for calculating the sample size in methodological studies of validity and reliability for the purpose of scale adaptations. The most widely used of these is based on the ratio of the number of study participants to the number of items. Hatcher and Gorsuch (Gorsuch, 2014) recommend a ratio of 5:1, while Nunnally (Thorndike, 1995) suggests a ratio of 10:1. Comrey and Lee’s rating scale for adequate sample sizes has been set forth as 50 = very poor, 100 = poor, 200 = fair, 300 = good, 500 = very good, and 1000 = excellent (Comrey & Lee, 1992). We recruited 317 participants for our study; 17 respondents were excluded from the study because they had missing data in three or more items.

### 2.3. Measures

The first part of the questionnaire consisted of a form that queried the participants’ sociodemographic characteristics; the second section comprised the 10-item Turkish version of the CARE measure.

#### 2.3.1. Demographic Questionnaire

This questionnaire was prepared based on a scan of the literature and comprises 8 items. The questions are related to the participants’ age, gender, civil status, the number of people in their household, their education, income perception, examination frequency, chronic diseases, and other similar demographics.

#### 2.3.2. The Consultation and Relational Empathy (CARE) Measure

The CARE measure was developed by Mercer, S.W., Maxwell, M., Heaney, D., and Watt, G.C.M. in 2004 (Mercer, 2004). The CARE measure is a 5-point Likert-type scale for which responses to its 10 items are scored on a range of “Poor” (1 point) to “Excellent” (5 points). All of the items can also be marked “Not applicable”. The overall scale score is found by multiplying the average scores of the items by 10. The maximum possible score on the scale is 50; the minimum is 10. The scale consists of only one factor, and there are no reversely scored items. Mercer et al. report that up to 2 “Not applicable” responses given by any participant or unanswered items yield results similar to missing data on the questionnaire (Mercer et al., 2005).

### 2.4. Data Analysis

The IBM SPSS Statistics (v.21) and Lisrel 8.7 programs were used for data analysis. Prior to the analysis, the scores were converted into z-scores, and a one-way scan was performed for outliers. No value outside of the cutoff value of ±3 was found in the scan for outliers, and thus, no data from the dataset were excluded from the analysis.

Face validity, “Not applicable” responses, and missing data were checked and evaluated. Cronbach’s alpha and McDonald’s omega reliability coefficients were used to test internal reliability. In addition, corrected item–total correlations were used to assess homogeneity.

To collect evidence on the measure’s construct validity, exploratory and confirmatory factor analyses were performed. Prior to the factor analyses, the Keiser–Meyer–Olkin (KMO) coefficient was computed to see whether the data were suitable for factor analysis. The exploratory and confirmatory analyses were then carried out to determine construct validity. In the evaluation of the results of the confirmatory factor analysis, the chi-square statistic (2, 2/sd), the Comparative Fit Index (CFI), Root Mean Square of Error Approximation (RMSEA), Non-Normed Fit Index (NNFI), Normed Fit Index (NFI), and Standardized Root Mean Square Residual (SRMR) goodness of fit indices were studied. The cut-off points used for the evaluation of the results of the confirmatory factor analysis are as follows (Erkorkmaz et al., 2013): CFI ≥ 0.95 indicates an excellent fit, RMSEA ≤ 0.08 indicates a good fit, RMSEA ≤ 0.05 indicates an excellent fit, NNFI ≥ 0.95 indicates an excellent fit, SRMR ≤ 0.08 indicates a good fit, and SRMR ≤0.05 indicates an excellent fit. ANOVA and the *t*-test were used in the comparison of the participants’ demographic characteristics and their total CARE measure scores.

### 2.5. Ethical Considerations

Permission for the conduct of the study was obtained from the Akdeniz University Faculty of Medicine Clinical Studies Ethics Committee (557/21.09.2022), and the written and verbal informed consent of all of the study participants was collected. The study was conducted in accordance with the principles of the Declaration of Helsinki.

## 3. Results

The mean age of the participants was 39.88 (SD: 14.95). Among the participants, 29.3% were aged 18–30 (n = 88), 36.3% were aged 31–43 (n = 109), 18.0% were aged 44–56 (n = 54), and 16.3% were aged 57 years and over (n = 49). It was noted that women formed the majority of the participants (58.7%; n = 176). The majority of the participants were married (65.7%; n = 197), while 27.7% (n = 83) were unmarried, and 6.7% (n = 20) were divorced/widowed. The demographic characteristics of the participants can be seen in Table 1.

The responses of the participants to the CARE questionnaire are summarized in Table 2 along with corresponding percentages. The mean CARE score was 36.47 (SD = 10.08; n = 300), which was observed to be within the general range of 12–50. Fifteen percent of the participants received the highest score of 50, and the lowest score of 12 in the study was displayed by 1.0% of total participants.

The fact that there were no missing data in our study and no responses of “Not applicable” indicates excellent face validity (Table 2). In terms of internal reliability, we found that Cronbach’s alpha coefficient was 0.973 for 10 items on the CARE measure, and McDonald’s omega coefficient was 0.973, indicating high reliability. When any one of the items on the CARE measure was deleted, Cronbach’s alpha and McDonald’s omega coefficients showed only a slight decrease. All corrected item–total correlations were in the range of 0.817–0.917 (Table 3).

Exploratory and confirmatory factor analyses were performed to assess construct validity. Prior to the exploratory factor analysis, the Keiser–Meyer–Olkin (KMO) coefficient was calculated as 0.954, indicating that the data structure was suitable for factor analysis. The exploratory factor analysis showed a variance of 78.42% and that the measure could be explained with a single factor with factor loadings varying between 0.829 and 0.931 (Table 3).

**Figure 1:**
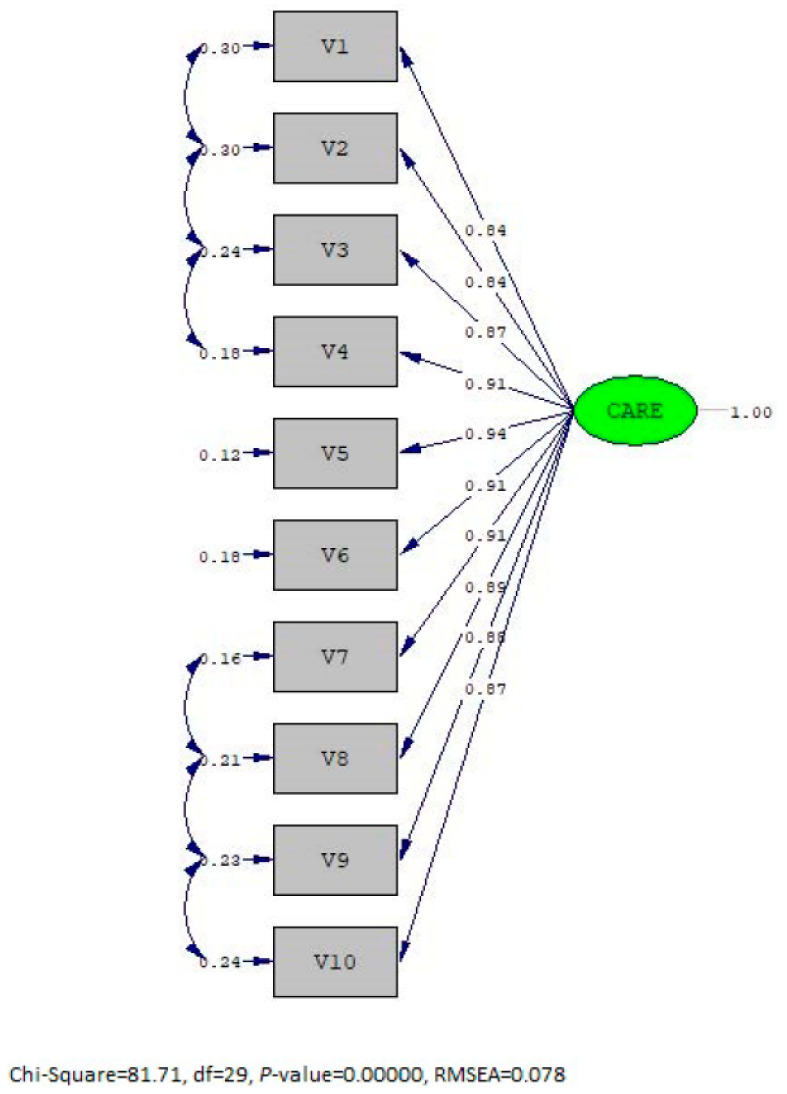
v10 represents questions of the measure. The order of elements is the same as in Table 2.

Confirmatory factor analysis was additionally performed to assess construct validity. The values indicating the goodness of fit with the model can be seen in Table 4. It was noted that the χ^2^/Sd value was less than three, RMSEA was less than 0.08, the CFI, NFI, and NNFI values were greater than 0.95, and SRMR was less than 0.05 (Table 4, Figure 1).

The total CARE score was examined to see whether it had been influenced by the participants’ demographic characteristics. We used ANOVA and a *t*-test for the examination and observed that the CARE score was not influenced by the patients’ gender (*p* = 0.612), age group (*p* = 0.801), civil status (*p* = 0.592), education level (*p* = 0.436), income (*p* = 0.541), examination frequency (*p* = 0.498), or the presence of a chronic disease (*p* = 0.400).

## 4. Discussion

In this study, we adapted the original English CARE measure to the Turkish language and tested this version for validity and reliability. When the frequency of the responses to the CARE measure was reviewed, we found, as was seen in studies carried out in other languages (Aomatsu et al., 2014; Crosta Ahlforn et al., 2017; Fung et al., 2009; García Del Barrio et al., 2021), that the answers leaned toward the responses “good”, “very good”, and “excellent”. A ceiling effect should be considered when the percentage of participants obtaining a ceiling score is above 15% (Terwee et al., 2007). We found in our study that 15% of our participants displayed the highest score of 50. Although the scores clustered around the higher levels, the fact that 15% of the participants displayed a maximum score of 50 and, further, that there was 0% at the lowest score of 10 was interpreted to mean that there was no ceiling effect. The studies conducted for Japanese and Chinese are similar, but although no ceiling effect could be seen in these languages (Aomatsu et al., 2014; Fung et al., 2009), a ceiling effect was observed in the Spanish (García Del Barrio et al., 2021), Swedish (Crosta Ahlforn et al., 2017), Brazilian Portuguese (Manzoni et al., 2019), Dutch (van Dijk et al., 2017), and original English (Mercer, 2004) versions. We noted that the mean total CARE score in versions where a ceiling effect had not been observed was lower than in those where a ceiling effect had been determined.

In terms of the reliability and validity of our study, our results were similar to previous adaptations of the study, as well as to the original study in which the measure was developed (Fung et al., 2009; García Del Barrio et al., 2021; Hanževački et al., 2015; Manzoni et al., 2019; Mercer, 2004; Natali et al., 2022; van Dijk et al., 2017). As in other adaptations of the measure, face validity was high (Aomatsu et al., 2014; Crosta Ahlforn et al., 2017; Fung et al., 2009; Hanževački et al., 2015; Mercer, 2004; Park et al., 2022; van Dijk et al., 2017). In terms of internal consistency, we found Cronbach’s alpha to be high (0.973), which was similar to the coefficients of 0.975 found in the Swedish version (Crosta Ahlforn et al., 2017), 0.953 in the Spanish version (García Del Barrio et al., 2021), and 0.962 in the Chinese version (Fung et al., 2009). The “if item deleted” value for Cronbach’s alpha for each of the 10 items was moderately lower than the total Cronbach’s alpha value, showing that all of the items made a positive contribution to internal consistency. The “if item deleted” value for Cronbach’s alpha in the versions of the measure in other languages showed a slight decrease (Aomatsu et al., 2014; Crosta Ahlforn et al., 2017; Fung et al., 2009; García Del Barrio et al., 2021; Hanževački et al., 2015; Manzoni et al., 2019; Park et al., 2022; Rajput et al., 2020; van Dijk et al., 2017). The item–total correlations, which indicated high homogeneity in the present study, were consistent with the adaptations in other languages (Aomatsu et al., 2014; Crosta Ahlforn et al., 2017; Fung et al., 2009; Natali et al., 2022). The results of the exploratory and confirmatory factor analyses yielded a single-factor structure. The confirmatory factor analysis indicated that the model represented a good and excellent goodness of fit. It can be seen that other language adaptations of the CARE measure also displayed a single-factor structure and high construct validity (Aomatsu et al., 2014; Crosta Ahlforn et al., 2017; Fung et al., 2009; García Del Barrio et al., 2021; Manzoni et al., 2019; Park et al., 2022; van Dijk et al., 2017).

The mean CARE score was 36.47 (SD = 10.08; n = 300). This score is similar to the mean score of the Japanese translation (Aomatsu et al., 2014) and slightly higher than the mean score of the Chinese translation (Fung et al., 2009). The mean scores of translations in European cultures are found to be slightly higher (García Del Barrio et al., 2021; Manzoni et al., 2019; Mercer et al., 2005; van Dijk et al., 2017). Larger and multi-centered cross-cultural studies are needed to explore the factors causing the differences in mean CARE scores between Asian and European cultures. Similarly to previous adaptations of the CARE measure in other languages, in this study, there was no need to add or remove items to scale.

In our comparison of our participants’ demographic data with the overall CARE score, we saw that the CARE score was not affected by the patients’ gender, age group, civil status, education, income level, examination frequency, or the presence of a chronic disease. It was noted that the CARE score was not reported to be affected by the patients’ demographic characteristics in the previous Japanese and Spanish (Aomatsu et al., 2014; García Del Barrio et al., 2021) versions either.

### Strengths and Weaknesses

The method of translation and adaptation of the original CARE measure that we used in our study was one of its strengths. The adaptation involved a translation and back-translation process that focused on producing an exact and culturally meaningful Turkish version of the measure. Another important strength was that both exploratory and confirmatory analyses were performed to evaluate construct validity. An additional strength was that the measure was implemented by the researchers and not the consulting physicians. This averted any bias that might have occurred if doctors had been asked to respond to the questionnaire. Reaching a wide sample was also a strength of the study. At the same time, creating a suitable space for patients where they did not feel that pressure was being placed on them during the data collection phase resulted in curbing the instances of missing data and “not applicable” responses.

The fact that our study was conducted at a single site, a tertiary care facility, was one of its weaknesses. Since we did not compare the performance of the CARE measure with any other empathy scale, we did not include additional questions to assess the patients’ general satisfaction with their consultation. This may be considered a weakness of the study.

The sample in this study does not represent the entire Turkish population or all patients at Akdeniz University Hospital. This can be considered a weakness of the study. It is important to underline that this study was designed to translate the CARE measure into Turkish and test its validity and reliability. Thus, the sample did not need to be representative of the entire Turkish population. Further studies with more data are needed to establish reference points.

## 5. Conclusions

The Turkish version of the CARE measure has high internal consistency and construct validity. The Turkish version can be used as a tool for patients to assess the empathy levels of their physicians. Because the Turkish CARE measure is in a similar format to the original CARE measure and its counterparts in other languages, it can be safely used in multi-site cross-cultural studies.

Further studies are required to establish reference values of the measure in the Turkish language and culture. Larger studies are warranted to determine whether the CARE measure can be used to determine the impact of interventions aiming to improve the empathy levels of physicians. In addition, larger datasets representing the entire Turkish population are needed to establish normative values for benchmarking.

## Figures and Tables

**Table 1:** Sociodemographic data of 300 individuals who presented for an examination from 1 October 2022 to 30 November 2022.

		n (%)
**Gender**	Female	176 (58.7%)
	Male	124 (41.3%)
**Age**	18–30	88 (29.3%)
	31–43	109 (36.4%)
	44–56	54 (18.0%)
	57 and over	49 (16.3%)
**Civil status**	Single	83 (27.7%)
	Married	197 (65.7%)
	Divorced/Widowed	20 (6.6%)
**Education**	Primary and lower	23 (7.6%)
	Middle School	32 (10.7%)
	High school	80 (26.7%)
	University or higher	165 (55.0%)
**Income Level**	Low	54 (18.0%)
	Average	230 (76.7%)
	High	16 (5.3%)
Examination frequency *	Once a year or less	56 (18.7%)
	2–6 times a year	144 (48.0%)
	7–12 times a year	49 (16.3%)
	13 times a year or more often	51 (17.0%)
Presence of chronic disease *	Yes	121 (40.3%)
	No	179 (59.7%)
Number of people in household *	1–4 people	244 (81.3%)
	5–10 people	56 (18.7%)

* Participant’s own declaration.

**Table 2:** CARE response pattern of 300 individuals who had presented for an examination from 1 October 2022 to 30 November 2022.

Care Measure Questions	Poor	Fair	Good	Very Good	Excellent	Not Answered or Missing	Total
1-Did you feel comfortable?	7 (2.3%)	39 (13.0%)	101 (33.7%)	81 (27.0%)	72 (24.0%)	0	300 (100%)
2-Were you given a chance to relate your story?	5 (1.7%)	26 (8.7%)	90 (30.0%)	93 (31.0%)	86 (28.7%)	0	300 (100%)
3-Did they really listen to you?	12 (4.0%)	32 (10.7%)	81 (27.0%)	96 (32.0%)	79 (26.3%)	0	300 (100%)
4-Did they show a holistic interest in you?	13 (4.3%)	39 (13.0%)	76 (25.3%)	86 (28.7%)	86 (28.7%)	0	300 (100%)
5-Did they fully understand your concerns?	18 (6.0%)	39 (13.0%)	74 (24.7%)	91 (30.3%)	78 (26.0%)	0	300 (100%)
6-Did they show you interest and compassion?	23 (7.7%)	39 (13.0%)	78 (26.0%)	89 (29.7%)	71 (23.7%)	0	300 (100%)
7-Did they have a positive approach towards you?	9 (3.0%)	31 (10.3%)	76 (25.3%)	99 (33.0%)	85 (28.3%)	0	300 (100%)
8-Were their explanations clear?	13 (4.3%)	35 (11.7%)	71 (23.7%)	90 (30.0%)	91 (30.3%)	0	300 (100%)
9-Did they help you arrange for a follow-up appointment?	13 (4.3%)	30 (10.0%)	75 (25.0%)	96 (32.0%)	86 (28.7%)	0	300 (100%)
10-Did they set up an action plan with you?	16 (5.3%)	38 (12.7%)	74 (24.7%)	85 (28.3%)	87 (29.0%)	0	300 (100%)

**Table 3:** Internal reliability, homogeneity, and factor loadings of the CARE measure (n = 300).

	Corrected Item–Total Correlation If Item Deleted	Cronbach’s α If Item Deleted	McDonald’s ω If Item Deleted	Factor Loading
C1	0.817	0.972	0.972	0.931
C2	0.838	0.971	0.971	0.911
C3	0.870	0.970	0.970	0.907
C4	0.893	0.969	0.970	0.897
C5	0.917	0.968	0.969	0.890
C6	0.884	0.970	0.970	0.884
C7	0.898	0.969	0.969	0.883
C8	0.877	0.970	0.970	0.867
C9	0.870	0.970	0.970	0.850
C10	0.855	0.971	0.971	0.829

C1–C10 represent questions of the measure. The order of questions is the same as in Table 2.

**Table 4:** Confirmatory factor analysis results for the CARE measure (n = 300).

	χ^2^	SD	χ^2^/SD	RMSEA	CFI	NFI	NNFI
Model	81.71	29	2.82	0.078	0.99	0.99	0.99

Abbreviations: χ^2^/SD: Chi-square statistic, CFI: Comparative Fit Index, RMSEA: Root Mean Square of Error Approximation, NNFI: Non-Normed Fit Index, NFI: Normed Fit Index.

## Data Availability

All data will be provided by the corresponding author upon request.

## Abbreviations

The following abbreviations are used in this manuscript:

CARE	Consultation and Relational Empathy
DSM-5	Diagnostic and Statistical Manual of Mental Disorders, fifth edition
CVI	Content validity index
KMO	Keiser–Meyer–Olkin coefficient
CFI	Comparative Fit Index
RMSEA	Root Mean Square of Error Approximation
NNFI	Non-Normed Fit Index
NFI	Normed Fit Index
SRMR	Standardized Root Mean Square Residual
ANOVA	Analysis of variance
